# Neutralizing-antibody response to SARS-CoV-2 for 12 months after the COVID-19 workplace outbreaks in Japan

**DOI:** 10.1371/journal.pone.0273712

**Published:** 2022-08-30

**Authors:** Azumi Ishizaki, Xiuqiong Bi, Quynh Thi Nguyen, Tomomi Maeno, Akinori Hara, Hiroyuki Nakamura, Sanae Kuramoto, Koichi Nishi, Hiroyasu Ooe, Hiroshi Ichimura

**Affiliations:** 1 Department of Viral Infection and International Health, Graduate School of Medical Science, Kanazawa University, Kanazawa, Japan; 2 Kaga Toshiba Electronics, Ishikawa, Japan; 3 Nexco, Ishikawa, Japan; 4 Department of Public Health, Graduate School of Medical Science, Kanazawa University, Kanazawa, Japan; 5 Ishikawa Prefectural Institute of Public Health and Environmental Science, Kanazawa, Japan; 6 Ishikawa Prefectural Central Hospital, Kanazawa, Japan; 7 Department of Laboratory, Kanazawa University Hospital, Kanazawa, Japan; Public Health England, UNITED KINGDOM

## Abstract

This study aimed to elucidate the 12-month durability of neutralizing antibodies (NAbs) against severe acute respiratory syndrome coronavirus 2 (SARS-CoV-2) in patients infected during the 2020 workplace outbreaks of coronavirus disease 2019 (COVID-19) in Japan. We followed 33 Japanese patients infected with SARS-CoV-2 in April 2020 for 12 months (12M). Patients were tested for NAbs and for antibodies against the SARS-CoV-2 nucleocapsid (anti-NC-Ab) and antibodies against the spike receptor-binding domain (anti-RBD-Ab). Tests were performed at 2M, 6M, and 12M after the primary infection (*api*) with commercially available test kits. In 90.9% (30/33) of patients, NAbs persisted for 12M *api*, though the median titers significantly declined from 78.7% (interquartile range [IQR]: 73.0–85.0%) at 2M, to 59.8% (IQR: 51.2–77.9) at 6M (*P* = 0.008), and to 56.2% (IQR: 39.6–74.4) at 12M (*P*<0.001). An exponential decay model showed that the NAb level reached undetectable concentrations at 35.5 months *api* (95% confidence interval: 26.5–48.0 months). Additionally, NAb titers were significantly related to anti-RBD-Ab titers (rho = 0.736, *P*<0.001), but not to anti-NC-Ab titers. In most patients convalescing from COVID-19, NAbs persisted for 12M *api*. This result suggested that patients need a booster vaccination within one year *api*, even though NAbs could be detected for over two years *api*. Anti-RBD-Ab titers could be used as a surrogate marker for predicting residual NAb levels.

## Introduction

Coronavirus disease 2019 (COVID-19), caused by severe acute respiratory syndrome coronavirus 2 (SARS-CoV-2), emerged in December 2019, and has caused a global pandemic [1]. Humoral immune responses, particularly antibodies specific to the receptor-binding domain (RBD) in the spike protein of SARS-CoV-2, play an important role in viral neutralization and clearance [2]. Several longitudinal cohort studies of patients with COVID-19 from China, the US, and Thailand have shown that neutralizing antibody (NAb) titers reached a peak around one month post-symptom onset, then declined, but they remained detectable for 12–13 months (12–13 M) post-symptom onset in over 95% of patients convalescing from COVID-19 [3–6].

Previous studies of four seasonal coronaviruses showed that humoral immunities against the viruses were short-lasting, and re-infections by the same coronavirus could occur within an interval of 12 M [7, 8]. SARS-CoV-2 re-infections were reported in 11%–16% of patients convalescing from COVID-19 within 6 M after the primary infection in large, population-based studies in the UK and US [9, 10]. To understand the long-term kinetics of humoral immune responses to primary SARS-CoV-2 infections, it is essential to evaluate the risk of re-infection and determine the appropriate timing for booster vaccinations, if necessary, to prevent re-infections.

This study aimed to elucidate the durability of antibodies against SARS-CoV-2, including antibodies against the nucleocapsid protein (anti-NC-Ab), antibodies against the RBD in the spike protein (anti-RBD-Ab), and neutralizing antibodies (NAbs). The test cohort included individuals infected with SARS-CoV-2 during the COVID-19 workplace outbreaks, which occurred in Ishikawa prefecture, Japan, in April 2020.

## Methods

### Study population and schedule for specimen collections

We recruited employees that acquired confirmed SARS-CoV-2 infections during the two outbreaks of COVID-19, which occurred at two workplaces in Ishikawa, Japan, in April 2020 (index cases). SARS-CoV-2 infections were confirmed with reverse transcription polymerase chain reaction (RT-PCR) tests for detecting viral RNA. We also recruited “close contacts”, defined as individuals who had been in close contact with an index case without taking necessary infection prevention measures, such as wearing a mask, practicing social distancing, and so on. Close contacts were identified by the contact-tracing surveillance team of the local government in Ishikawa Prefecture, Japan, and included all household members of the index cases. Close contacts underwent RT-PCR tests for SARS-CoV-2, based on the circumstances of their contact with the index case(s) and their clinical symptoms [11]. All household members of the index case underwent RT-PCR tests.

Blood specimens were collected from index patients between June and July 2020, 2–3 M after the COVID-19 outbreak in the workplace. Individuals that showed positive anti-NC-Ab results at 2 M were followed at 6 M (i.e., in November and December 2020) and at 12 M (i.e., in April 2021) after the workplace outbreaks.

### Laboratory procedures

After blood collection, sera were separated by centrifugation at 3000 × g for 10 min and stored at −80°C until use. Anti-NC-Ab and anti-RBD-Ab were quantified with electrochemiluminescence immunoassays, Elecsys® Anti-SARS-CoV-2 (S300) RUO and Elecsys® Anti-SARS-CoV-2 S (S300) RUO (Roche Diagnostics, IN, USA), respectively. These assays were performed with a fully automated Cobas 8000 Analyzer Series Module e801 (Roche Diagnostics). Results are expressed in terms of a cutoff index (COI) or a cutoff concentration. The presence of anti-NC-Ab was positive when the detection value was ≥1.0 COI, and the presence of anti-RBD-Ab was positive when the detection value was ≥0.8 U/mL. The ability of NAbs to inhibit SARS-CoV-2 binding to its target was quantified, in duplicate, with an enzyme-linked immunosorbent assay (ELISA)-based SARS-CoV-2 Surrogate Virus neutralization Test (sVNT) Kit (GenScript, Jiangsu, China) [12]. The cutoff level (lower limit of quantification) for the positive detection of NAbs was set at 30% inhibition, according to manufacturer instructions.

### Statistical analysis

Statistical analyses were performed with SPSS version 25 (SPSS Japan Inc., Tokyo, Japan). The chi-squared test or Fisher’s exact test and Mann-Whitney *U* test were performed to assess differences between groups. Changes in antibody levels were compared between paired samples with the Friedman test, and the Bonferroni correction was applied in post hoc analyses. Spearman’s rank correlation coefficient was used to assess correlations between the levels of anti-NC-Ab, anti-RBD-Ab, and NAbs. A receiver operating characteristic (ROC) curve was plotted to evaluate the sensitivity and specificity of anti-RBD-Abs to predict the NAb level. Simple linear regression analysis was conducted to test associations between the levels of antibodies at 2 M, 6 M, and 12 M, and relevant variables, including age, sex, comorbidity, and disease severity. The NAb decay rate was calculated with an exponential decay model [13]. *P*-values <0.05 were considered significant.

### Ethical approval

This study was carried out according to the World Medical Association’s Declaration of Helsinki and the Japanese Ethics Guidelines for Human Genome/Gene Analysis Research. The study protocol was approved by the ethics committee of Kanazawa University (3388–1). All participants were appropriately informed about the study, and all provided written consent to participate in this study. When a participant under 18 years old agreed to participate, consent was obtained from the parents or guardians.

## Results

### Study participants

We recruited 135 company employees, including 27 index cases (21 males and 6 females; median age: 48.0 years, interquartile range [IQR]: 41.0‒54.0; Table 1) and 108 close contacts (86 males and 22 females; median age: 49.0 years, IQR: 42.3‒54.8). The sex and age distributions of participants did not significantly differ between the index cases and close contacts (*P* = 0.832 and *P* = 0.684, respectively). We also included 11 household members of the index cases (2 males and 9 females; median age: 46.0 years, IQR: 14.0‒67.0). None of the participants had received the COVID-19 vaccine or exhibited any clinical symptom suggestive of COVID-19 during the follow-up period.

**Table 1:** Gender and age distribution of the participants and their SARS-CoV-2 PCR and anti-NC-Ab results at 2M.

	N	Sex Male (%)	Age Median, IQR (years)	Anti-NC-Ab positive at 2M, n (%)
Index cases (RT-PCR positive)	27	21 (77.8)	48.0 (41.0‒54.0)	27 (100%)
Closed contact colleagues (RT-PCR negative or not tested)	108	86 (79.6)^1^	49.0 (42.3‒54.8)^2^	5 (4.6%)
Close contact household members	11	2 (18.2)	46.0 (14.0‒67.0)	3 (27.3%)
RT-PCR positive	3	1 (33.3)	46.0 (11.0‒)	3 (100%)
RT-PCR negative	8	1 (12.5)	49.5 (16.0‒74.5)	0 (0%)

RT-PCR: reverse transcription polymerase chain reaction for severe acute respiratory syndrome coronavirus 2 (SARS-CoV-2); IQR: interquartile range; Anti-NC-Ab: antibody to the nucleocapsid of SARS-CoV-2; 2M: 2 to 3 months after the COVID-19 outbreak in the workplace; *P*-values between index cases versus close contacts are based on the Mann-Whitney *U* test or Fisher’s exact test; 1: *P* = 0.832; 2: *P* = 0.684.

### Antibodies at 2‒3 months after the COVID-19 workplace outbreaks

All 27 index cases showed positive results for the anti-NC-Ab. Among the 108 close contacts, five (4.6%) showed positive results for the anti-NC-Ab (Table 1). These five close contacts had not undergone RT-PCR testing during the workplace outbreaks. Of the 11 household members, three (27.3%) had sustained SARS-CoV-2 infections, confirmed by RT-PCR, and all three showed positive anti-NC-Ab results. Thus, a total of 35 participants had been infected with SARS-CoV-2 during the outbreaks. Additionally, all of these 35 participants showed positive anti-RBD-Ab and NAb results.

### Changes in anti-NC-Ab levels at 6 and 12 months after the outbreaks

Among the 35 individuals with positive anti-NC-Ab results, 33 agreed to additional follow-ups at 6 and 12 M. Of the 33, three were asymptomatic, 27 had mild symptoms, and the remaining three had moderate symptoms. Among these 33 individuals, 33 (100%) and 32 (97.0%) showed positive anti-NC-Ab results at 6 and 12 M, respectively (S1 Table).

**Fig 1:**
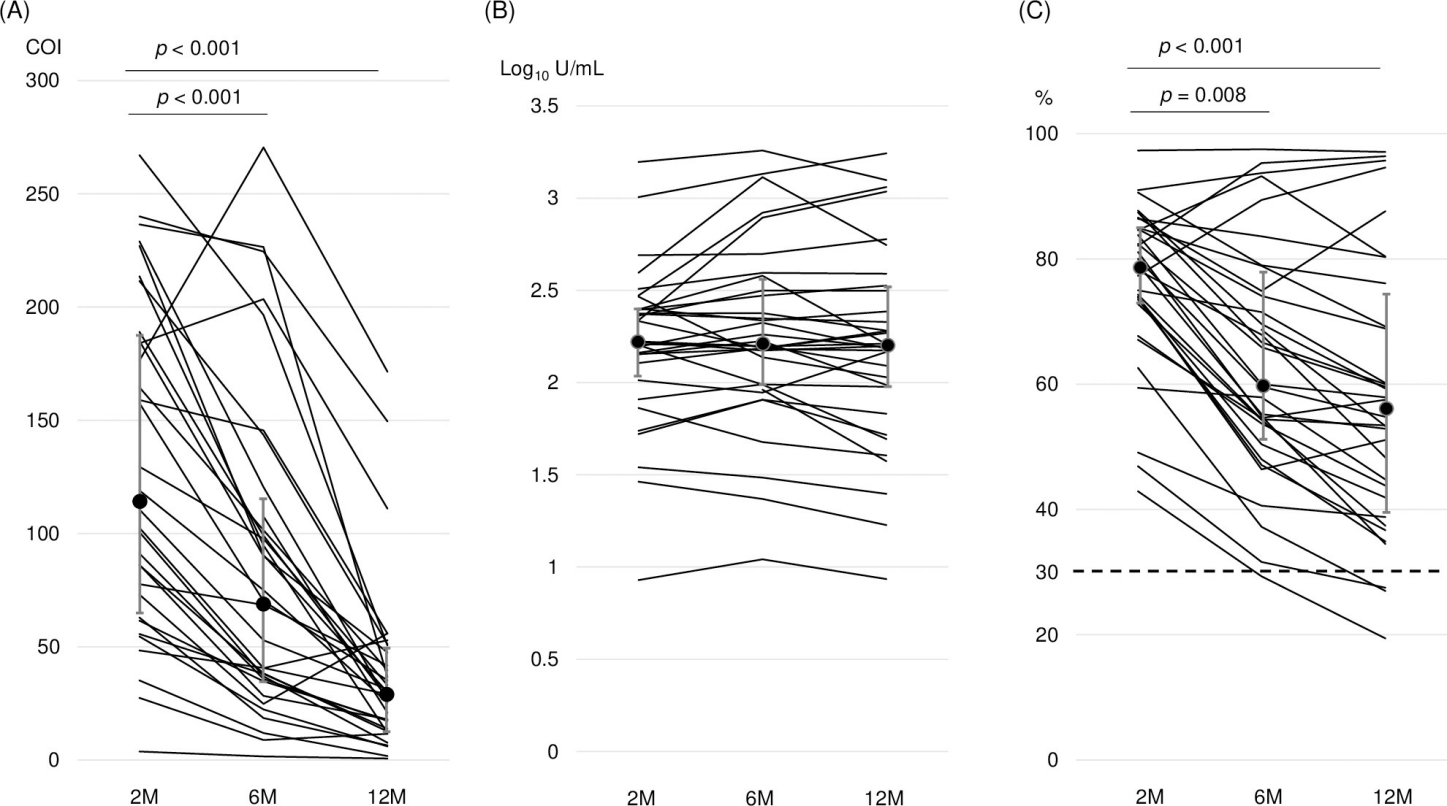
The changes of antibody responses from 2M, 6M, to 12M after the outbreaks of COVID-19 at workplaces among 33 with asymptomatic, mild to moderate COVID-19. (A) The changes of the anti-nucleocapsid antibody. Cutoff index (COI) ≧ 1.0 is positive. (B) The changes of the anti-receptor-binding domain antibody. The numeric value ≥ 0.8 U/mL [Log_10_(0.8) = ‒0.0969] is positive. (C) The changes of the inhibition rate of antibody specific to the receptor-binding domain that is equivalent to neutralizing antibody. The positive cutoff is ≧ 30%, indicated as the dotted line. Significance among three points was analyzed by the Friedman test and post hoc analysis with a Bonferroni correction. The dot and error bars correspond to the median for anti-nucleocapsid antibody and anti-receptor-binding domain and the median titer for inhibition rate of neutralizing antibody ± interquartile range.

The anti-NC-Ab titers declined significantly from a median COI of 114.8 (IQR: 65.4–188.0) at 2 M, to a median COI of 69.4 (IQR: 35.0–115.8) at 6 M and a median COI of 29.5 (IQR: 12.9–49.8) at 12 M (2M vs. 6M, *P*<0.001; 2 M vs. 12 M, *P*<0.001; 6 M vs. 12 M, *P* = 0.001; Fig 1A).

### Changes in anti-RBD-Ab levels at 6 and 12 months after the outbreaks

All 33 participants included in 12-M follow-ups showed positive anti-RBD-Ab results at both 6 and 12 M. The anti-RBD-Ab titers did not significantly change over time. The median anti-RBD-Ab titers were (log_10_ U/mL) 2.2 (IQR: 2.0–2.4) at 2 M, 2.2 (IQR: 2.0–2.6) at 6 M, and 2.2 (IQR: 2.0–2.5) at 12 M (*P* = 0.14 among the three groups; Fig 1B).

### Changes in NAb levels at 6 and 12 months after the outbreaks

Of the 33 participants followed, 32 (97.0%) and 30 (90.9%) showed positive NAb results at 6 and 12 M, respectively. The median NAb titer significantly declined over time, with 78.7% inhibition (IQR: 73.0–85.0%) at 2 M, 59.8% (IQR: 51.2–77.9) at 6 M (*P* = 0.008), and 56.2% (IQR: 39.6–74.4) at 12 M (2 M vs. 12 M, *P*<0.001; 6 M vs. 12 M, *P* = 0.026; Fig 1C).

In three individuals, NAb titer levels declined from 2 to 6 M, then rebounded at 12 M. In the other three individuals, NAb titer levels increased steadily, from 2 to 12 M. However, in these six participants, the anti-NC-Ab titer levels decreased constantly over the same observation periods (Fig 1B and Table 2). In one participant, the anti-NC-Ab result was negative at 12 M, but the anti-RBD-Ab and NAb results remained positive at 12 M (Table 2).

**Table 2:** The changes of the anti-NC-Ab, anti-RBD-Ab, and NAb among the individuals whose NAb titer increased from 6M to 12M and whose anti-NC-Ab turned to be negative.

ID	Sex	Age	Comorbidity	Severity	Anti-NC-Ab (COI)			Anti-RBD-Ab (Log_10_ U/mL)			NAb (%)		
					2M	6M	12M	2M	6M	12M	2M	6M	12M
NAb re-increased at 12M than 6M													
24	M	38	No	Mild	267	196.5	50.6	2.01	1.94	2.17	74	46.4	51.1
25	M	49	No	Mild	110.5	52.9	31.2	2.11	2.19	2.28	67.1	54.6	57.5
26	M	11	No	Mild	164.5	101.5	39.6	2.69	2.70	2.78	86.6	74.9	87.6
NAb increased in 6-12M than 2M													
27	M	56	Yes	Mild	91.1	35.7	17.2	2.34	2.89	3.04	82.1	95.3	96.4
28	F	32	No	Mild	61.5	34.7	17.7	2.47	2.92	3.06	77.3	89.4	94.6
29	M	39	No	Mild	240	224.5	149.5	3.01	3.13	3.24	91	93.7	95.7
Anti-NC-Ab became negative													
30	M	52	No	Mild	3.7	1.5	0.6	1.91	1.99	1.98	49.1	40.6	38.8

Anti-NC-Ab: anti-nucleocapsid antibody, ≥ 1.0 COI (cut-off index) is positive; Anti-RBD-Ab: anti-receptor binding domain antibody, ≥ ‒0.0969 Log_10_ U/mL (≥ 0.8 U/ml) is positive; NAb: neutralizing antibody, ≥ 30% (% of inhibition) is positive; 2M: 2 to 3 months after the COVID-19 outbreak in the workplace; 6M: 6 months after the outbreak; 12M: 12 months after the outbreak; Mild: no pneumonia; Moderate: pneumonia not requiring oxygen.

### Correlation between the NAb and anti-RBD-Ab titers

**Fig 2:**
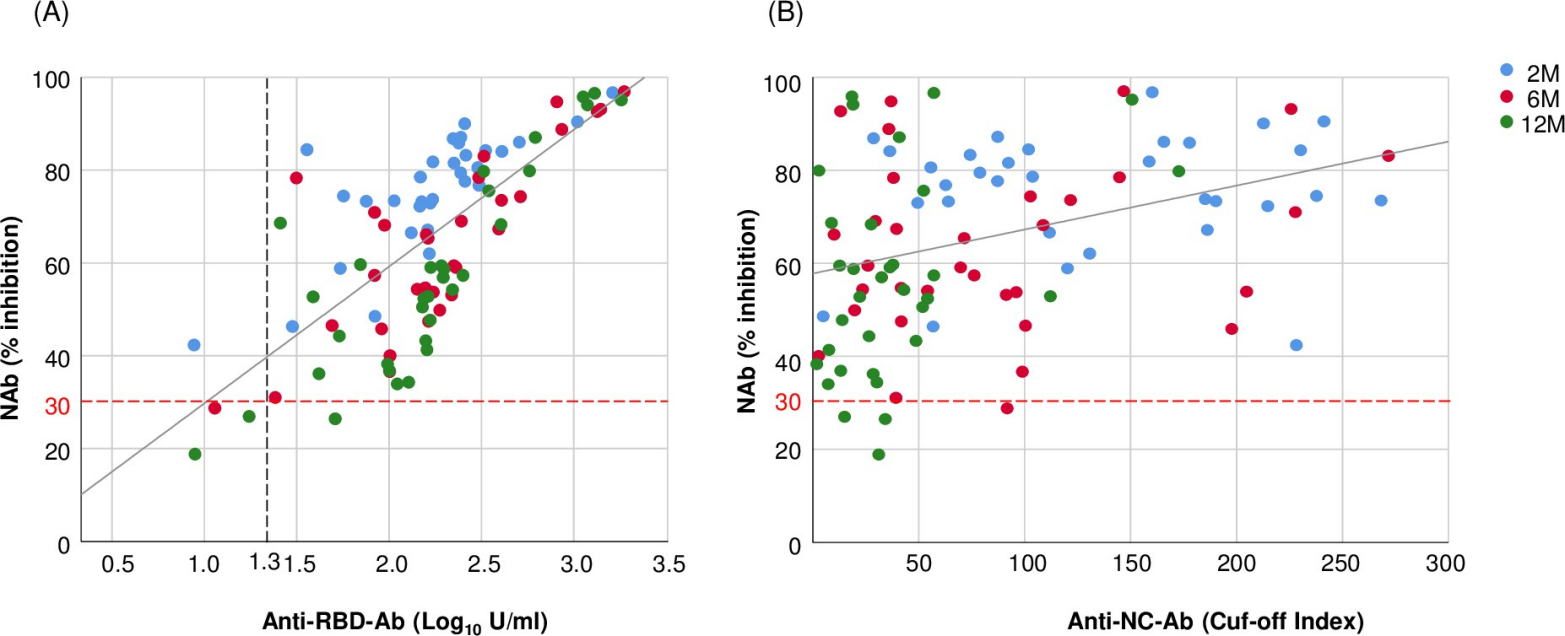
Correlations of the neutralizing antibody (NAb) with anti-RBD-Ab and anti-NC-Ab. (A) Correlation between the NAb and the anti-receptor-binding domain antibody (anti-RBD-Ab). The cutoff for anti-RBD-Ab, Log_10_ (0.8) = ‒0.0969, is not shown in the graph. Spearman’s rank correlation coefficient: 2M: rho = 0.739; 6M: rho = 0.707; 12M: rho = 0.801; in total: rho = 0.736; all *P* < 0.001. Blue dots: data at 2 months after the outbreaks of COVID-19 at workplaces (2M); red dots: data at 6 months (6M); and green dots: data at 12 months (12M). The solid line represents the linear regression. The red dotted line indicates the cutoff of NAb. The black dotted line indicates the suggested threshold of anti-RBD-Ab to determine the positivity of NAb. (B) Correlation between the NAb and the anti-nucleocapsid antibody (anti-NC-Ab). The cutoff for anti-NC-Ab is Cut off index (COI) ≧ 1.0. Spearman’s rank correlation coefficient: 2M: rho = 0.062, *p* = 0.73; 6M: rho = 0.129, *p* = 0.48; 12M: rho = 0.268, *p* = 0.13; in total: rho = 0.355, *P* < 0.001.

NAb titers were strongly correlated with the anti-RBD-Ab titers at all time points (rho = 0.739 at 2 M; rho = 0.707 at 6 M; rho = 0.801 at 12 M; all *P*<0.001) and over the entire study period (rho = 0.736, *P*<0.001; Fig 2A). The ROC curve analysis indicated an anti-RBD-Ab cutoff threshold of 1.3 Log_10_ U/mL for the ability of anti-RBD-Ab to predict NAb positivity, with a sensitivity of 98.9% and a specificity of 75.0%.

The NAb titers were not significantly correlated with anti-NC-Ab titers at any time point (rho = 0.062 at 2 M, *P* = 0.73; rho = 0.129 at 6 M, *P* = 0.48; and rho = 0.268 at 12 M, *P* = 0.13). However, over the entire study period, NAb titers were mildly correlated with anti-NC-Ab titers (rho = 0.355; *P*<0.001; Fig 2B). Other factors, including age, sex, disease severity, and comorbidity, were not significantly associated with the titers of anti-NC-Ab, anti-RBD-Ab, or NAb, at any time point (P>0.05 for all analyses, S2–S4 Tables).

### Prediction of NAb durability

**Fig 3:**
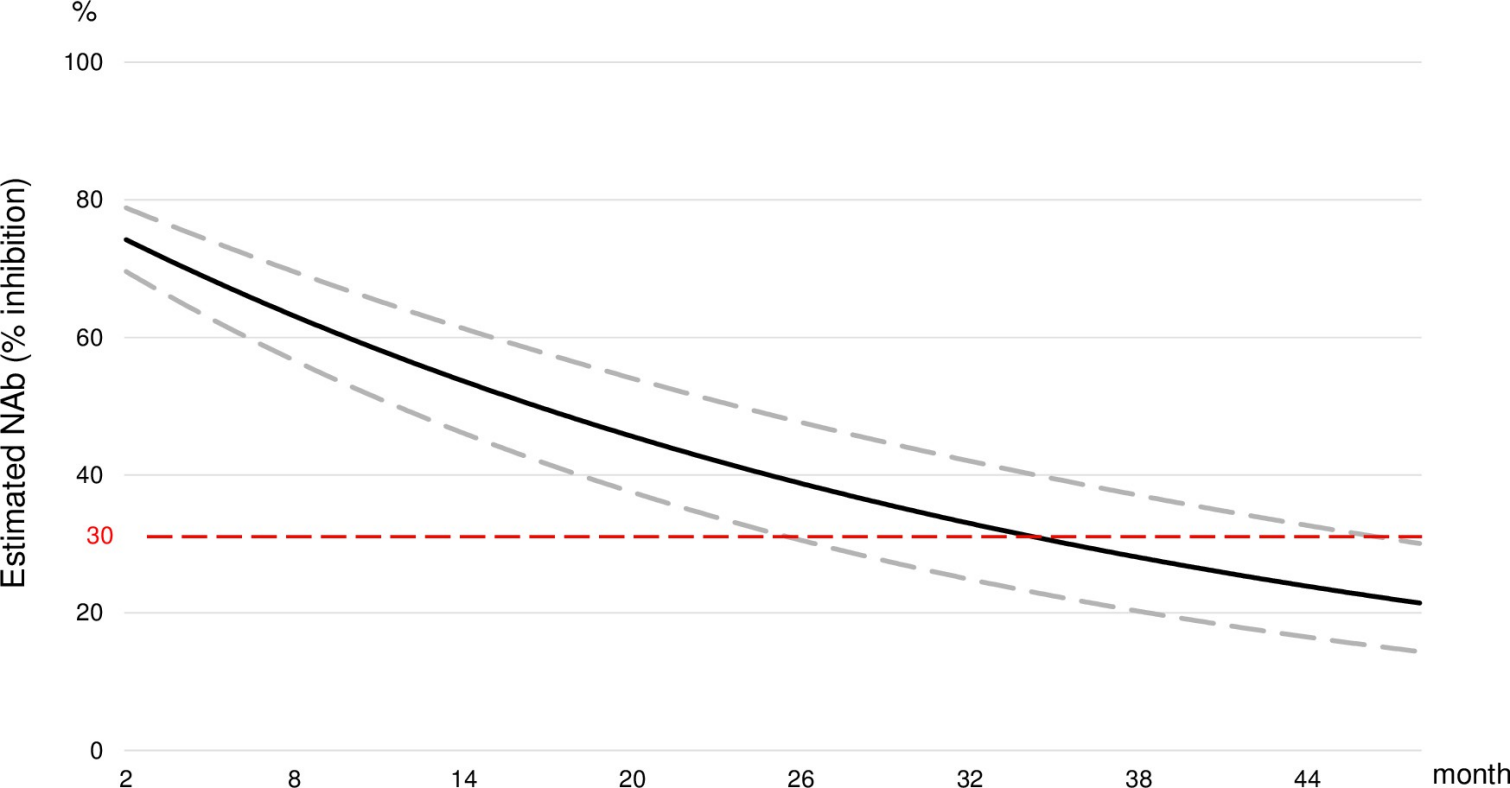
The estimated decay curve of best fit for neutralizing antibody (NAb). The solid line indicates the estimated level of the NAb and dotted lines indicate the upper and the lower bounds of the 95% confidence intervals. The red dotted line indicates the cutoff line for 30% of NAb.

We estimated that the NAb level declined to undetectable values at about 35.5 months (95% confidence interval [CI]: 26.5–48.0 months) after the outbreak (Fig 3).

## Discussion

In this study, we analyzed the 12-month durability of NAb titers in 33 individuals with first-time SARS-CoV-2 infections that displayed zero to moderate symptoms in Japan. We found that 90.9% (30/33) of these individuals maintained NAbs for 12 months, although NAb titers had declined significantly at 12 M, regardless of age, sex, comorbidity, and disease severity.

Plasma NAb levels are known to be correlated with the prevention of SARS-CoV-2 infections and COVID-19 onset, and/or the reduction of disease severity. However, to date, no consensus has been established on the specific titer needed to prevent infection [14]. Townsend *et al*. estimated that, after a primary infection, the likely time interval for a SARS-CoV-2 re-infection was between 3 months and 5.1 years, with a median of 16 months [15]. Nguyen *et al*. reported that the SARS-CoV-2 delta variant was capable of breakthrough infections among fully vaccinated Vietnamese health care workers. In those workers, the median NAb titer determined with the sVNT was 59.4% (IQR: 32.5–73.1%) at diagnosis [16]. Our exponential decay model showed that the NAb level would decline to undetectable levels (30% inhibition) at 35.5 months (95% CI: 26.5–48) after a SARS-CoV-2 infection, and that NAb levels would fall to 59.4% inhibition at 10.3 months (95% CI: 6.6–15.5) after the primary infection. Because a decline in NAbs increases the susceptibility to a breakthrough infection [17], our findings suggested that individuals convalescing from COVID-19 should receive a booster vaccination within one year after the primary infection to prevent re-infections [5, 18–20].

Our results also showed that NAb titers at 2, 6, and 12 M did not correlate with disease severity. Several previous studies reported that the magnitude of the peak NAb titer depended on disease severity during early convalescence [6, 21–23]. However, studies on the differences between severity groups in NAb titers measured 3 to 12 M after symptom onset have produced discrepant results [4, 6, 22, 23]. These discrepancies could be due to differences in the target populations. For example, our study did not include severe cases, but other studies included severe and critical cases. Alternatively, the discrepancies could be due to differences in the methods for detecting NAbs. In the present study, we used the sVNT, but other studies used neutralizing assays with pseudotyped virus and/or authentic virus. Importantly, all four longitudinal cohort studies with observation periods of 12–13 M showed that NAb titers declined over time [3–6]. Thus, booster vaccinations should be recommended for all patients convalescing from COVID-19, regardless of disease severity.

We found that NAb titers were strongly correlated with anti-RBD-Ab titers at each time point and over the entire study period (all rho>0.7; *P*<0.001). Our ROC curve analysis indicated that anti-RBD-Ab titers ≥1.3 Log_10_ U/mL (equivalent to ≥20 U/ml), could predict NAb positivity with a sensitivity of 98.9% and specificity of 75.0%. The anti-RBD-Ab assay utilized in the current study was fully automated, and the run-time was short, compared to the run-time for the manual sVNT assay. Considering these findings, the anti-RBD-Ab titer could be a useful alternative marker for predicting NAb positivity and for monitoring the level of herd immunity in a community.

In six participants of our cohort, the anti-RBD-Ab and NAb levels increased from 2 M to 6 and 12 M. However, the anti-NC-Ab titers continued to decline over the same period. None of these participants reported clinical COVID-19 symptoms during the study period. Some previous longitudinal studies also reported an increase in NAb in patients convalescing from COVID-19 during 6 to 12 M after disease onset, but no mechanism has been proposed [4, 24]. We speculate that this continuous humoral response after the convalescent period could be induced by re-exposure to SARS-CoV-2, by the persistence of spike proteins, or by cross-reactivity to other human seasonal coronaviruses. In the current study, several SARS-CoV-2 endemics had occurred in the area during the study period. Thus, a re-exposure to SARS-CoV-2 without triggering an infection might have boosted the anti-RBD-Ab and NAb titers without increasing the anti-NC-Ab titer in six individuals in our study. Additionally, Gaebler *et al*. reported that persistent SARS-CoV-2 antigens were detected in individuals convalescing from COVID-19; viral antigen detection persisted for an average of 4 M after disease onset [25]. Additionally, antibodies against the S proteins of seasonal human coronavirus were shown to be boosted by a SARS-CoV-2 infection [26, 27]. Therefore, the anti-SARS-CoV-2 antibodies might have been induced by cross-reactivity to other seasonal human coronavirus infections [28]. In this study, we did not conduct RT-PCR tests to detect SARS-CoV-2 RNA during observation period, since none of the participants exhibited any clinical symptom suggestive of COVID-19 and testing capacity for COVD-19 RT-PCR in Japan was limited during the study period, nor did we investigate viral antigens or antibodies to other human coronaviruses in individuals convalescing from COVID-19. Further investigations are needed to understand the underlying mechanisms of prolonged humoral responses in patients convalescing from COVID-19.

The present study showed that, during the COVID-19 outbreaks at workplaces, 4.6% of close contacts were missed in diagnosing COVID-19. To control the epidemic in Japan, more active surveillance is needed to identify all COVID-19 cases, including asymptomatic cases. Moreover, we observed secondary attacks in 27.3% of households; this rate was slightly higher than those reported previously (6 to 16.6%) in systematic reviews on household SARS-CoV-2 transmission [29, 30]. The Japanese national whole-genome epidemiologic surveillance project demonstrated that the SARS-CoV-2 strain B.1.1. variant caused the epidemic that occurred in late March through April 2020 in Japan, including the area sampled in the present study [31]. In contrast, the omicron variants were the predominant circulating strain in the latest epidemic in Japan. The omicron variant showed higher transmissibility within households than previously circulating variants [32]. Thus, more caution and stronger measures to prevent household transmission are needed in Japan.

The current study has several limitations. First, the small number of COVID-19 cases included could have limited the significance of the findings. Second, none of the study participants had the opportunity to receive COVID-19 vaccination during the observation period, from June 2020 to April 2021, since the COVID-19 vaccination program for the general population in Japan began in June 2021. Recently, however, it was reported that 58 individuals with SARS-CoV-2 infection history in the same area of the current study, including 19 of the current study participants, had a median anti-RBD-Ab titer of 4.2 log_10_ U/mL [range 2.8 to 5.0] and NAbs titers of more than 95% after two doses of the vaccines [33]. This recent report would support our suggestion that a booster vaccination within one year after the primary infection will be beneficial, considering a median anti-RBD-Ab titer of 2.2 log_10_ U/mL (IQR: 2.0–2.5) and a median NAbs titer of 56.2% (IQR: 39.6–74.4) at 12M in the current study. Third, we utilized a surrogate virus neutralization test. This ELISA was based on antibody-mediated blockage of the interaction between the angiotensin-converting enzyme 2 receptor protein and the viral RBD [12]. However, a titration of the neutralizing antibody against an authentic virus requires biosafety level (BSL) 3 facilities. Alternatively, the sVNT assay was reported to be comparable with the virus neutralization test against an authentic virus and/or pseudo-type virus [12]. Thus, we adopted the sVNT, which is simple and can be performed in a BSL2 laboratory.

Currently, infections by new variants in convalescing or vaccinated individuals is a growing concern for COVID-19 containment [34–36]. Therefore, in future, we must also evaluate the sera in participants that were infected with the B.1.1. variant to determine the neutralizing capacity against emerging variants. That evaluation will require an authentic or pseudo-type virus-based assay.

## Conclusion

Our findings suggested that patients convalescing from a primary SARS-CoV-2 infection should receive a COVID-19 booster vaccination within one year after the infection. We also showed that the anti-RBD-Ab could be used as a surrogate marker to predict the residual level of NAbs. Our findings provided new insight into the durability of humoral immune responses to SARS-CoV-2 and suggested an appropriate timing for booster vaccinations in patients convalescing from COVID-19.

## Supporting information

S1 Table Details of the antibody titers. (DOCX)

S2 Table Simple linear regression analysis of anti-NC-Ab with age, sex, comorbidity, and disease severity at 2M, 6M, and 12M. (DOCX)

S3 Table Simple linear regression analysis of anti-RBD-Ab with age, sex, comorbidity, and disease severity at 2M, 6M, and 12M. (DOCX)

S4 Table Simple linear regression analysis of NAb with age, sex, comorbidity, and disease severity at 2M, 6M, and 12M. (DOCX)

## References

[1] Lu R, Zhao X, Li J, et al. Genomic characterisation and epidemiology of 2019 novel coronavirus: implications for virus origins and receptor binding. Lancet. 2020;395(10224):565–74. doi: 10.1016/S0140-6736(20)30251-8 32007145 PMC7159086

[2] Poland GA, Ovsyannikova IG, Kennedy RB. SARS-CoV-2 immunity: review and applications to phase 3 vaccine candidates. Lancet. 2020;396(10262):1595–606. doi: 10.1016/S0140-6736(20)32137-1 33065034 PMC7553736

[3] Shi D, Weng T, Wu J, et al. Dynamic Characteristic Analysis of Antibodies in Patients With COVID-19: A 13-Month Study. Front Immunol. 2021;12:708184. doi: 10.3389/fimmu.2021.708184 34354712 PMC8330131

[4] Zhang J, Lin H, Ye B, et al. One-year sustained cellular and humoral immunities of COVID-19 convalescents. Clin Infect Dis. 2021. published online Oct 5. doi: 10.1093/cid/ciab884 34609506 PMC8524303

[5] Wang Z, Muecksch F, Schaefer-Babajew D, et al. Naturally enhanced neutralizing breadth against SARS-CoV-2 one year after infection. Nature. 2021;595(7867):426–31. doi: 10.1038/s41586-021-03696-9 34126625 PMC8277577

[6] Vacharathit V, Srichatrapimuk S, Manopwisedjaroen S, et al. SARS-CoV-2 neutralizing antibodies decline after one year and patients with severe COVID-19 pneumonia display a unique cytokine profile. Int J Infect Dis. 2021. published online Sep 15. 10.1016/j.ijid.2021.09.021. PMC8442529 34536610

[7] Edridge AWD, Kaczorowska J, Hoste ACR, et al. Seasonal coronavirus protective immunity is short-lasting. Nat Med. 2020;26(11):1691–3. doi: 10.1038/s41591-020-1083-1 32929268

[8] Sariol A, Perlman S. Lessons for COVID-19 Immunity from Other Coronavirus Infections. Immunity. 2020;53(2):248–63. doi: 10.1016/j.immuni.2020.07.005 32717182 PMC7359787

[9] Hall VJ, Foulkes S, Charlett A, et al. SARS-CoV-2 infection rates of antibody-positive compared with antibody-negative health-care workers in England: a large, multicentre, prospective cohort study (SIREN). Lancet. 2021;397(10283):1459–69. doi: 10.1016/S0140-6736(21)00675-9 33844963 PMC8040523

[10] Lumley SF, O’Donnell D, Stoesser NE, et al. Antibody Status and Incidence of SARS-CoV-2 Infection in Health Care Workers. N Engl J Med. 2021;384(6):533–40. doi: 10.1056/NEJMoa2034545 33369366 PMC7781098

[11] Furuse Y, Ko YK, Saito M, et al. Epidemiology of COVID-19 Outbreak in Japan, from January-March 2020. Jpn J Infect Dis. 2020;73(5):391–3. doi: 10.7883/yoken.JJID.2020.271 32350228

[12] Tan CW, Chia WN, Qin X, et al. A SARS-CoV-2 surrogate virus neutralization test based on antibody-mediated blockage of ACE2-spike protein-protein interaction. Nat Biotechnol. 2020;38(9):1073–8. doi: 10.1038/s41587-020-0631-z 32704169

[13] Whitcombe AL, McGregor R, Craigie A, et al. Comprehensive analysis of SARS-CoV-2 antibody dynamics in New Zealand. Clin Transl Immunology. 2021;10(3):e1261. doi: 10.1002/cti2.1261 33747511 PMC7955949

[14] Khoury DS, Cromer D, Reynaldi A, et al. Neutralizing antibody levels are highly predictive of immune protection from symptomatic SARS-CoV-2 infection. Nat Med. 2021;27(7):1205–11. doi: 10.1038/s41591-021-01377-8 34002089

[15] Townsend JP, Hassler HB, Wang Z, et al. The durability of immunity against reinfection by SARS-CoV-2: a comparative evolutionary study. Lancet Microbe. 2021. published online Oct 1. doi: 10.1016/S2666-5247(21)00219-6 34632431 PMC8486316

[16] Nguyen VVC, Nghiem MN, Lam NA, et al. An observational study of breakthrough SARS-CoV-2 Delta variant infections among vaccinated healthcare workers in Vietnam. EClinicalMedicine. 2021;41:101143. doi: 10.1016/j.eclinm.2021.101143 34608454 PMC8481205

[17] Bergwerk M, Gonen T, Lustig Y, et al. Covid-19 Breakthrough Infections in Vaccinated Health Care Workers. N Engl J Med. 2021;385(16):1474–84. doi: 10.1056/NEJMoa2109072 34320281 PMC8362591

[18] Fabricius D, Ludwig C, Scholz J, et al. mRNA Vaccines Enhance Neutralizing Immunity against SARS-CoV-2 Variants in Convalescent and ChAdOx1-Primed Subjects. Vaccines (Basel). 2021;9(8). doi: 10.3390/vaccines9080918 34452043 PMC8402682

[19] Ebinger JE, Fert-Bober J, Printsev I, et al. Antibody responses to the BNT162b2 mRNA vaccine in individuals previously infected with SARS-CoV-2. Nat Med. 2021;27(6):981–4. doi: 10.1038/s41591-021-01325-6 33795870 PMC8205849

[20] Jahrsdörfer B, Fabricius D, Scholz J, et al. BNT162b2 Vaccination Elicits Strong Serological Immune Responses Against SARS-CoV-2 Including Variants of Concern in Elderly Convalescents. Front Immunol. 2021;12:743422. doi: 10.3389/fimmu.2021.743422 34659239 PMC8511403

[21] Seow J, Graham C, Merrick B, et al. Longitudinal observation and decline of neutralizing antibody responses in the three months following SARS-CoV-2 infection in humans. Nat Microbiol. 2020;5(12):1598–607. doi: 10.1038/s41564-020-00813-8 33106674 PMC7610833

[22] Crawford KHD, Dingens AS, Eguia R, et al. Dynamics of neutralizing antibody titers in the months after SARS-CoV-2 infection. J Infect Dis. 2021;223(2):197–205. 33535236 10.1093/infdis/jiaa618 PMC7543487

[23] Pradenas E, Trinité B, Urrea V, et al. Stable neutralizing antibody levels 6 months after mild and severe COVID-19 episodes. Med (N Y). 2021;2(3):313–20.e4. doi: 10.1016/j.medj.2021.01.005 33554155 PMC7847406

[24] Chia WN, Zhu F, Ong SWX, et al. Dynamics of SARS-CoV-2 neutralising antibody responses and duration of immunity: a longitudinal study. Lancet Microbe. 2021;2(6):e240–e249. doi: 10.1016/S2666-5247(21)00025-2 33778792 PMC7987301

[25] Gaebler C, Wang Z, Lorenzi JCC, et al. Evolution of antibody immunity to SARS-CoV-2. Nature. 2021;591(7851):639–44. doi: 10.1038/s41586-021-03207-w 33461210 PMC8221082

[26] Dispinseri S, Secchi M, Pirillo MF, et al. Neutralizing antibody responses to SARS-CoV-2 in symptomatic COVID-19 is persistent and critical for survival. Nat Commun. 2021;12(1):2670. doi: 10.1038/s41467-021-22958-8 33976165 PMC8113594

[27] Anderson EM, Goodwin EC, Verma A, et al. Seasonal human coronavirus antibodies are boosted upon SARS-CoV-2 infection but not associated with protection. Cell. 2021;184(7):1858–64.e10. doi: 10.1016/j.cell.2021.02.010 33631096 PMC7871851

[28] Ng KW, Faulkner N, Cornish GH, et al. Preexisting and de novo humoral immunity to SARS-CoV-2 in humans. Science. 2020;370(6522):1339–43. doi: 10.1126/science.abe1107 33159009 PMC7857411

[29] Li F, Li YY, Liu MJ, et al. Household transmission of SARS-CoV-2 and risk factors for susceptibility and infectivity in Wuhan: a retrospective observational study. Lancet Infect Dis. 2021; 21(5):617–28. doi: 10.1016/S1473-3099(20)30981-6 33476567 PMC7833912

[30] Ng OT, Marimuthu K, Koh V, et al. SARS-CoV-2 seroprevalence and transmission risk factors among high-risk close contacts: a retrospective cohort study. Lancet Infect Dis. 2021;21(3):333–43. doi: 10.1016/S1473-3099(20)30833-1 33152271 PMC7831879

[31] Sekizuka T, Itokawa K, Hashino M, et al. A Genome Epidemiological Study of SARS-CoV-2 Introduction into Japan. mSphere. 2020;5(6). doi: 10.1128/mSphere.00786-20 33177213 PMC7657588

[32] Baker JM, Nakayama JY, O’Hegarty M, et al. SARS-CoV-2 B.1.1.529 (Omicron) Variant Transmission Within Households—Four U.S. Jurisdictions, November 2021–February 2022. MMWR Morb Mortal Wkly Rep 2022;71:341–6. doi: 10.15585/mmwr.mm7109e1 35238860 PMC8893332

[33] Bi X, Takayama T, Tokoro M, et al. Longer intervals before vaccination increase spike antibody titers in individuals previously infected with SARS-CoV-2. Microbiol Spectr. 2022 Apr 27;10(2):e0023822. doi: 10.1128/spectrum.00238-22 35384720 PMC9045212

[34] Garcia-Beltran WF, Lam EC, St Denis K, et al. Multiple SARS-CoV-2 variants escape neutralization by vaccine-induced humoral immunity. Cell. 2021;184(9):2372–83.e9. doi: 10.1016/j.cell.2021.03.013 33743213 PMC7953441

[35] Edara VV, Hudson WH, Xie X, Ahmed R, Suthar MS. Neutralizing Antibodies Against SARS-CoV-2 Variants After Infection and Vaccination. JAMA. 2021;325(18):1896–8. doi: 10.1001/jama.2021.4388 33739374 PMC7980146

[36] Cromer D, Juno JA, Khoury D, et al. Prospects for durable immune control of SARS-CoV-2 and prevention of reinfection. Nat Rev Immunol. 2021; 21(6):395–404. doi: 10.1038/s41577-021-00550-x 33927374 PMC8082486

