# Supplementary material for: Neutralizing-antibody response to SARS-CoV-2 for 12 months after the COVID-19 workplace outbreaks in Japan
Source: PLoS One. 2022 Aug 30;17(8):e0273712. doi: 10.1371/journal.pone.0273712 (PMC9426944; doi:10.1371/journal.pone.0273712)
Supplement: S1 Table — (DOCX) [file pone.0273712.s001.docx]

**S1 Table** **Details of the antibody titers**

| ID | Sex | Age | Comorbidity | Severity | Anti-NC-Ab (COI) | | | Anti-RBD-Ab (Log_10_ U/mL) | | | NAb (%) | | |
| --- | --- | --- | --- | --- | --- | --- | --- | --- | --- | --- | --- | --- | --- |
|  |  |  |  |  | 2M | 6M | 12M | 2M | 6M | 12M | 2M | 6M | 12M |
| 1 | M | 53 | No | Asymptomatic | 100.6 | 36.9 | 7.6 | 1.54 | 1.48 | 1.40 | 85.0 | 78.9 | 69.2 |
| 2 | F | 56 | No | Mild | 73.0 | 24.8 | 55.9 | 2.40 | 2.34 | 2.39 | 83.8 | 60.0 | 57.9 |
| 3 | F | 51 | No | Mild | 77.6 | 68.5 | 41.7 | 2.37 | 2.35 | 2.33 | 80.0 | 59.6 | 54.8 |
| 4 | M | 48 | No | Mild | 54.5 | 22.3 | 6.0 | 2.47 | 2.14 | 2.03 | 81.1 | 54.9 | 34.5 |
| 5 | M | 54 | Yes | Mild | 86.1 | 28.3 | 18.0 | 2.37 | 2.38 | 2.28 | 87.7 | 69.6 | 59.3 |
| 6 | F | 46 | No | Mild | 185.0 | 90.0 | 47.5 | 2.19 | 2.32 | 2.18 | 67.7 | 53.7 | 43.8 |
| 7 | M | 45 | No | Mild | 189.0 | 99.1 | 27.3 | 1.86 | 1.68 | 1.61 | 73.9 | 47.1 | 36.7 |
| 8 | F | 41 | No | Mild | 62.8 | 18.5 | 6.4 | 2.16 | 2.26 | 2.19 | 73.8 | 50.4 | 41.9 |
| 9 | M | 40 | No | Mild | 213.5 | 94.8 | 12.0 | 2.15 | 2.23 | 1.99 | 72.8 | 54.3 | 37.4 |
| 10 | F | 48 | No | Mild | 48.3 | 40.6 | 29.0 | 2.21 | 2.20 | 2.09 | 73.5 | 48.0 | 34.9 |
| 11 | F | 38 | No | Mild | n.d. | 107.5 | 20.9 | n.d. | 1.96 | 1.57 | n.d. | 68.7 | 53.3 |
| 12 | M | 56 | No | Mild | 102.5 | 40.5 | 52.9 | 2.16 | 2.18 | 2.17 | 79.1 | 55.2 | 52.9 |
| 13 | M | 44 | No | Moderate | 236.5 | 226.5 | 36.6 | 1.74 | 1.91 | 1.83 | 75.0 | 71.5 | 60.2 |
| 14 | M | 46 | No | Moderate | 229.0 | 120.5 | 26.3 | 2.51 | 2.59 | 2.59 | 84.8 | 74.1 | 68.9 |
| 15 | M | 44 | No | Mild | 86.0 | 38.3 | 12.6 | 2.40 | 2.58 | 2.21 | 78.2 | 67.9 | 48.3 |
| 16 | M | 63 | Yes | Asymptomatic | 176.5 | 270.5 | 171.5 | 2.36 | 2.50 | 2.50 | 86.4 | 83.6 | 80.3 |
| 17 | M | 50 | Yes | Moderate | 27.4 | 8.8 | 11.5 | 2.33 | 2.19 | 2.27 | 87.4 | 66.7 | 60.0 |
| 18 | M | 55 | No | Mild | 157.5 | 70.2 | 35.2 | 2.22 | 2.20 | 2.21 | 82.4 | 65.9 | 59.6 |
| 19 | M | 49 | No | Mild | 184.0 | 203.5 | 111.0 | 2.22 | 2.17 | 2.20 | 74.3 | 54.4 | 53.4 |
| 20 | M | 57 | No | Mild | 211.5 | 143.5 | 51.1 | 2.39 | 2.47 | 2.53 | 90.6 | 79.0 | 76.1 |
| 21 | M | 59 | No | Mild | 159.0 | 145.5 | 55.9 | 3.20 | 3.26 | 3.10 | 97.3 | 97.5 | 97.1 |
| 22 | M | 52 | No | Mild | 35.1 | 11.9 | 1.7 | 2.60 | 3.11 | 2.75 | 84.6 | 93.2 | 80.4 |
| 23 | F | 46 | No | Mild | 119.0 | 75.0 | 25.3 | 1.72 | 1.91 | 1.72 | 59.4 | 57.9 | 44.8 |
| 24 | M | 38 | No | Mild | 267.0 | 196.5 | 50.6 | 2.01 | 1.94 | 2.17 | 74.0 | 46.4 | 51.1 |
| 25 | M | 49 | No | Mild | 110.5 | 52.9 | 31.2 | 2.11 | 2.19 | 2.28 | 67.1 | 54.6 | 57.5 |
| 26 | M | 11 | No | Mild | 164.5 | 101.5 | 39.6 | 2.69 | 2.70 | 2.78 | 86.6 | 74.9 | 87.6 |
| 27 | M | 56 | Yes | Mild | 91.1 | 35.7 | 17.2 | 2.34 | 2.89 | 3.04 | 82.1 | 95.3 | 96.4 |
| 28 | F | 32 | No | Mild | 61.5 | 34.7 | 17.7 | 2.47 | 2.92 | 3.06 | 77.3 | 89.4 | 94.6 |
| 29 | M | 39 | No | Mild | 240.0 | 224.5 | 149.5 | 3.01 | 3.13 | 3.24 | 91.0 | 93.7 | 95.7 |
| 30 | M | 52 | No | Mild | 3.7 | 1.5 | 0.6 | 1.91 | 1.99 | 1.98 | 49.1 | 40.6 | 38.8 |
| 31 | M | 28 | No | Asymptomatic | 227.0 | 90.5 | 29.9 | 0.93 | 1.04 | 0.93 | 42.9 | 29.3 | 19.4 |
| 32 | M | 46 | No | Mild | 55.6 | 38.0 | 13.8 | 1.46 | 1.37 | 1.23 | 46.9 | 31.6 | 27.5 |
| 33 | M | 46 | No | Asymptomatic | 129.5 | 97.7 | 33.0 | 2.20 | 1.99 | 1.69 | 62.6 | 37.2 | 27.0 |

Anti-NC-Ab: anti-nucleocapsid antibody, ≥ 1.0 COI (cut-off index) is positive; Anti-RBD-Ab: anti-receptor binding domain antibody, ≥ ‒0.0969 Log_10_ U/mL (≥ 0.8 U/ml) is positive; NAb: neutralizing antibody, ≥ 30% (% of inhibition) is positive; 2M: 2 to 3 months after the COVID-19 outbreak in the workplace; 6M: 6 months after the outbreak; 12M: 12 months after the outbreak; n.d.: no data; Mild: no pneumonia; Moderate: pneumonia not requiring oxygen.
