# Supplementary material for: Neutralizing-antibody response to SARS-CoV-2 for 12 months after the COVID-19 workplace outbreaks in Japan
Source: PLoS One. 2022 Aug 30;17(8):e0273712. doi: 10.1371/journal.pone.0273712 (PMC9426944; doi:10.1371/journal.pone.0273712)
Supplement: S2 Table — (DOCX) [file pone.0273712.s002.docx]

**S2** **Table Simple linear regression analysis of anti-NC-Ab with age, sex, comorbidity, and disease severity at 2M, 6M, and 12M**

|  | 2M | | | 6M | | | 12M | | |
| --- | --- | --- | --- | --- | --- | --- | --- | --- | --- |
| Variable | Beta | SE | *P*-value | Beta | SE | *P*-value | Beta | SE | *P*-value |
| Age | -0.260 | 1.285 | 0.151 | -0.051 | 1.272 | 0.780 | 0.160 | 0.683 | 0.374 |
| Sex | -0.298 | 30.315 | 0.097 | -0.237 | 28.393 | 0.184 | -0.108 | 15.779 | 0.549 |
| Comorbidity | -0.196 | 39.299 | 0.309 | -0.025 | 39.745 | 0.897 | 0.146 | 21.345 | 0.442 |
| Disease severity | -0.010 | 28.135 | 0.957 | -0.050 | 27.218 | 0.781 | -0.226 | 14.418 | 0.206 |

Anti-NC-Ab: anti-nucleocapsid antibody; Beta: regression coefficient; SE: standard error; 2M: 2 to 3 months after the COVID-19 outbreak in the workplace; 6M: 6 months after the outbreak; 12M: 12 months after the outbreak.
