# Supplementary material for: Neutralizing-antibody response to SARS-CoV-2 for 12 months after the COVID-19 workplace outbreaks in Japan
Source: PLoS One. 2022 Aug 30;17(8):e0273712. doi: 10.1371/journal.pone.0273712 (PMC9426944; doi:10.1371/journal.pone.0273712)
Supplement: S3 Table — (DOCX) [file pone.0273712.s003.docx]

**S3 Table** **Simple linear regression analysis of anti-RBD-Ab with age, sex, comorbidity, and disease severity at 2M, 6M, and 12M**

|  | 2M | | | 6M | | | 12M | | |
| --- | --- | --- | --- | --- | --- | --- | --- | --- | --- |
| Variable | Beta | SE | *P*-value | Beta | SE | *P*-value | Beta | SE | *P*-value |
| Age | 0.141 | 0.008 | 0.441 | 0.131 | 0.009 | 0.469 | 0.006 | 0.112 | **0.010** |
| Sex | 0.011 | 0.188 | 0.951 | 0.026 | 0.203 | 0.884 | -0.008 | -0.006 | 0.220 |
| Comorbidity | 0.096 | 0.194 | 0.621 | 0.146 | 0.235 | 0.44 | 0.288 | 0.208 | 0.256 |
| Disease severity | 0.276 | 0.160 | 0.126 | 0.264 | 0.182 | 0.138 | 0.339 | 0.297 | 0.196 |

Anti-RBD-Ab: anti-receptor binding domain antibody; Beta: regression coefficient; SE: standard error; 2M: 2 to 3 months after the COVID-19 outbreak in the workplace; 6M: 6 months after the outbreak; 12M: 12 months after the outbreak; *P*-value in bold is significant.
