# Supplementary material for: Neutralizing-antibody response to SARS-CoV-2 for 12 months after the COVID-19 workplace outbreaks in Japan
Source: PLoS One. 2022 Aug 30;17(8):e0273712. doi: 10.1371/journal.pone.0273712 (PMC9426944; doi:10.1371/journal.pone.0273712)
Supplement: S4 Table — (DOCX) [file pone.0273712.s004.docx]

**S4 Table** **Simple linear regression analysis of NAb with age, sex, comorbidity, and disease severity at 2M, 6M, and 12M**

|  | 2M | | | 6M | | | 12M | | |
| --- | --- | --- | --- | --- | --- | --- | --- | --- | --- |
| Variable | Beta | SE | *P*-value | Beta | SE | *P*-value | Beta | SE | *P*-value |
| Age | 0.268 | 0.225 | 0.138 | 0.188 | 0.325 | 0.294 | 0.094 | 0.385 | 0.602 |
| Sex | -0.107 | 5.549 | 0.560 | -0.088 | 7.565 | 0.625 | -0.117 | 8.812 | 0.518 |
| Comorbidity | 0.312 | 6.139 | 0.1 | 0.313 | 9.303 | 0.092 | 0.282 | 10.921 | 0.131 |
| Disease severity | 0.246 | 4.792 | 0.174 | 0.171 | 6.978 | 0.340 | 0.158 | 8.169 | 0.379 |

NAb: neutralizing antibody; Beta: regression coefficient; SE: standard error; 2M: 2 to 3 months after the COVID-19 outbreak

in the workplace; 6M: 6 months after the outbreak; 12M: 12 months after the outbreak.
